# Evaluation of Trastuzumab Anti-Tumor Efficacy and its Correlation with HER-2 Status in Patient-Derived Gastric Adenocarcinoma Xenograft Models

**DOI:** 10.1007/s12253-015-9909-8

**Published:** 2015-03-09

**Authors:** Hao Chen, Qingqing Ye, Jing Lv, Peng Ye, Yun Sun, Shuqiong Fan, Xinying Su, Paul Gavine, Xiaolu Yin

**Affiliations:** 1 Department of General Surgery, Ren Ji Hospital, School of Medicine, Shanghai Jiao Tong University, No. 160 Pujian Road, Shanghai, 200127 China; 2 Department of Translational Science, Asia & Emerging Market iMed, AstraZeneca R&D, 199 Liangjing Road, Shanghai, 201203 China

**Keywords:** HER2, Gastric carcinoma, Trastuzumab, Xenograft model antitumor assays, Fluorescence in situ hybridization, Immunohistochemistry

## Abstract

The aim of the study was to investigate trastuzumab anti-tumor efficacy and its correlation with HER-2 status in primary xenograft models derived from Chinese patients with gastric adenocarcinoma. Patient-derived gastric adenocarcinoma xenograft (PDGAX) mouse models were firstly generated by implanting gastric adenocarcinoma tissues from patients into immune deficient mice. A high degree of histological and molecular similarity between the PDGAX mouse models and their corresponding patients’ gastric adenocarcinoma tissues was shown by pathological observation, *HER-2* expression, *HER-2* gene copy number, and mutation detection. Based on Hoffmann’s criteria in gastric cancer, three models (PDGAX001, PDGAX003 and PDGAX005) were defined as *HER-2* positive with fluorescence in situ hybridization (FISH) amplification or immunohistochemistry (IHC) 2+/ 3+, while two models (PDGAX002, PDGAX004) were defined as HER-2 negative. Upon trastuzumab treatment, significant tumor regression (105 % TGI) was observed in model PDGAX005 (*TP53* wt), while moderate sensitivity (26 % TGI) was observed in PDGAX003, and resistance was observed in PDGAX001, 002 and 004. A significant increase in *HER-2* gene copy number was only observed in PDGAX005 (*TP53* wt). Interestingly, trastuzumab showed no efficacy in PDGAX001 (HER2 IHC 3+ and FISH amplification, but with mutant *TP53*). Consistent with this finding, phosphor-HER2 modulation by trastuzumab was observed in model PDGAX005, but not in PDGAX001.

## Introduction

Gastric cancer (GC) is the second most common cancer and leading cause of cancer mortality worldwide [1–4]. Early stage patients with GC usually show no specific symptoms and as a consequence, GC patients often present with advanced disease upon diagnosis, especially in countries where gastric cancer screening is not routinely performed [4, 5].

Surgery remains the fundamental treatment option for resectable gastric cancer. However, even after curative surgery, local and distant recurrence rates are still high. Advanced-stage gastric cancer is associated with poor prognosis and low 5-year survival rates (5–20 %) [6]. Although adjuvant chemotherapy using epirubicin, cisplatin, and 5-flurouracil (5-FU) has gradually become standard-of-care therapy for GC patients in many countries [7], most patients with inoperable or metastatic disease require palliative treatment and have a median overall survival of less than 1 year and a limited chance of long-term survival [6, 8].

Due to recent genomic and proteomic advances and improved understanding of the key molecular pathways in cancer, molecular targeted therapies have emerged as additional treatment options in clinical practice. HER2 protein overexpression is increasingly recognized as a frequent molecular abnormality, known to be driven by *HER-2* gene amplification in breast cancer [9]. Overexpression of the HER2 protein and amplification of the *HER-2* gene in gastric cancer were first described in 1986 [10]. In recent years, HER-2 has gradually become a new prognostic factor and a novel therapeutic target in GC [9].

Trastuzumab is a recombinant humanized monoclonal antibody targeted against the HER2 extracellular domain [11]. The ‘Trastuzumab in gastric cancer’ (ToGA) study was the first randomized, controlled phase III trial to evaluate trastuzumab efficacy and safety in HER2-positive advanced gastric cancer [12]. In this trial, 22.1 % of the patients were identified as positive for HER-2 protein expression [8] and the median overall survival was significantly improved after trastuzumab treatment plus chemotherapy, compared to chemotherapy alone (13.8 *vs* 11.1 months) [13]. Importantly, trastuzumab showed no significant concomitant increase in treatment side effects [13], leading to its approval in 2010 in the EU and US for use in combination with 5-FU or capecitabine plus cisplatin for the first-line treatment of patients with HER2-positive metastatic adenocarcinoma of the stomach or GE junction [1, 5]. The European Medicines Agency recommended immunohistochemistry (IHC) as the first-line test for HER-2, with patients scoring 3+ eligible for trastuzumab therapy and 2+ cases classified as equivocal and necessitating a confirmatory fluorescence in situ hybridization (FISH) result [14]. The US FDA defined positivity by utilizing the eligibility criteria of the ToGA trial: IHC 3+ or 2+ plus FISH amplification [14]. However, unlike breast cancer [15], accurate and finalized HER-2 scoring criteria for gastric cancer patient selection still remains a subject for debate [16].

Herein we describe the generation and characterization of xenograft mouse models derived directly from patients’ gastric adenocarcinoma tissues. Furthermore, we use these novel clinically relevant models to evaluate the current HER-2 scoring system and to explore the opportunity for trastuzumab targeted therapy in gastric carcinoma patients.

## Materials and Methods

### Patients and Tissue Samples

Five treatment-naïve gastric adenocarcinoma patient samples were obtained intraoperatively during gastrectomy resection at the Renji Hospital, Shanghai, China. Freshly harvested gastric adenocarcinoma specimens from patient tumors were separated into three parts: the first part was transferred to medium-containing antibiotics immediately after surgical resection under sterile conditions, then transported to an animal facility within 2 h for implantation into immune deficient mice; the second part was snap frozen immediately in liquid nitrogen for DNA/ RNA extraction; and the third part was fixed in formalin and embedded in paraffin for pathological and IHC analysis. All the samples were firstly evaluated by pathologist for quality control purposes. Informed consent was obtained from all patients. This study was approved by the ethics board of Renji Hospital, Shanghai Jiaotong University.

### Establishment of Patient Derived Gastric Adenocarcinoma Xenograft (PDGAX) Mouse Models

Eight to 10 week old female nude (*nu/nu*) mice and severe combined immune deficient (SCID) mice (Vital River, Beijing, China) were used for the generation of xenograft models in this study. Animals were kept in a controlled light–dark cycle (12 h-12 h). The PDGAX mouse models were established from fresh patient gastric adenocarcinoma (GA) tissues surgically resected from GC patients. Patient’s GA tissues (F0 tissue) were cut into fragments of approximately 15 mm^3^ and implanted subcutaneously via Trocar needle into female SCID mice within 2 h after surgery. The patient tumor-engrafted mice were observed daily for 90 days. Once tumors started to grow, subcutaneous caliper measurements were taken weekly. Around 500 mm^3^ GA tumors was harvested from each tumor-bearing mice and further implanted in another batch of female nude mice. After three consecutive mouse-to-mouse passages, the xenograft was considered to be stable and submitted for full characterization, including histopathological analysis, HER-2 expression by FISH and IHC assays, and mutation detection of *AKT1, FGFR4, PIK3CA, PTCH, PTEN* and *TP53*. The tumor specimens in each passage of the tumor-bearing mice were harvested and divided into three parts. The first part was implanted into immune deficient mice for the next generation of the xenograft model; the second part was snap frozen in liquid nitrogen for DNA/RNA extraction; and the third part was fixed in 10 % buffered formalin for 24 h and embedded in paraffin for IHC analysis. Surplus fresh tumor tissues at passage 3–5 were frozen with 20 % FCS in liquid nitrogen for future model recovery. The PDGAX mouse models were maintained in nude mice within ten passages for efficacy studies. All animal experiments were performed in accordance with IACUC-approved guidelines.

### H&E Staining

All the xenograft and primary tissues were fixed in 10 % buffered formalin within 30 min after resection. Tissues were processed using a routine procedure after 24 h fixation. Sectioned slides were stained with hematoxylin and eosin, and then reviewed by pathologist to confirm the GA diagnosis.

### Immunohistochemistry

All incubations were performed at room temperature and all washing steps were performed with TBST. Tissues sectioned at 4 μm were firstly dewaxed and rehydrated. Antigen retrieval was performed using a pressure cooker at 110 °C for 5 min in retrieval buffer (S2367, DAKO). Endogenous peroxidase activity was blocked with 3 % hydrogen peroxide (S2023, DAKO). Sections were then incubated with HER-2 antibody (Herceptest^TM^, DAKO) for 30 min and phospho-HER2 (pHER2) antibody (1:600, Epitomics 2521–1) for 60 min, respectively. Finally, immunocomplexes were detected by incubation with Envision system (DAKO K5204) for 30 min and diaminobenzidine (K3468, DAKO) for 10 min.

In the present study, the IHC scoring criteria on human gastric cancer followed Hoffman’s criteria [16]: no staining or <10 % tumor cell positive staining was defined as 0/negative; faintly or barely perceptible staining on >10 % tumor cell membrane was defined as 1+; weak to moderate positive staining on >10 % tumor cells was defined as 2+; and cohesive moderate to strong staining on the membrane was defined as 3 + .

### Fluorescence in Situ Hybridization

HER2/CEP17 FISH probes were obtained from Vysis (Cat #30-161060) and FISH assays were performed as per the manufacturer’s instructions. In brief, TMA sections were dewaxed, dehydrated, and 10 μl HER2/CEP17 probes were applied. Sections were codenaturated together with probes at 75 °C for 4 min, then incubated overnight at 37 °C. Subsequently, probes were washed off twice sequentially with 0.3 % NP40/1 × SSC at 75.5 °C for 5 min, 2 × SSC at room temperature for 2 min, and then dehydrated and air-dried. Sections were mounted in 0.3 μg/ml DAPI mounting medium (diluted from Vector, Cat #H-1200), and stored at 4 °C (avoiding light) for at least 30 min prior to scoring. In each case, 50 tumor nuclei were evaluated. Intact interphase tumor nuclei identified by DAPI staining were evaluated, and gene (red signal) and CEP17 (green signal) copy numbers in each nucleus were assessed. An absolute HER-2 gene copy number lower than four or an HER-2/CEP17 ratio of less than 1.8 was considered HER-2 negative. HER-2 copy number between 4 and 6, or a HER-2/CEP17 ratio between 1.8 and 2.0 was considered HER-2 equivocal. Cases showing a tight gene cluster of HER-2 signals, an average gene copy number of HER-2 > 6, or a HER-2/CEP17 fluorescence ratio ≥ 2 were considered positive for gene amplification. The above criteria was based on the Hoffmann criteria in GC [16] and other investigations [17, 18].

### Trastuzumab Antitumor Efficacy Studies

For therapeutic experiments, suitable tumor-bearing, HER-2 positive mice in the tumor volume range of 150 to 250 mm^3^ were sorted randomly (8 animals per group) and assigned to vehicle control or trastuzumab treatment groups (Roche, China, 15 mg/kg/twice weekly intraperitoneally). Nude mouse subcutaneous tumor volumes and body weights were measured twice per week. Tumor volume was calculated from two perpendicular diameters using the formula: V = (length + [width]^2^) /2. The calculated volumes were standardized to the values at the first treatment day. Percentage of tumor growth inhibition (%TGI) was calculated with the formula [1 – (change of tumor volume in treatment group/change of tumor volume in control group)] × 100, which was used for the evaluation of anti-tumor efficacy.

### Screening of Gene Mutations

Formalin-fixed paraffin-embedded tumor blocks were reviewed for quality and tumor content. A single representative block, containing both PDGAX mouse model and the corresponding patient GA sample (each containing at least 70 % of neoplastic cells) was screened for gene mutations. Genomic DNA was extracted using the QIAamp Mini kit (Qiagen) as per the manufacturer’s instructions. ‘Hot spot’ mutations in *AKT1* (exon 18, 19, 20, 21), *FGFR4* (exon 2 and 3), *PIK3CA* (exon 10 and 15), *PTCH* (exon 1, 2), *PTEN* (exon 1,2) and *TP53* genes (exon 5 and 7) were screened using amplification refractory mutation system (ARMS, Amoy diagnostics, Fujian, China) and mutant-enriched liquid chip PCR method (SurExam Bio-Tech, Guangzhou, China) [19].

### Statistical Analysis

Statistical significance was evaluated using a one-tailed, two-sample Student’s *t* test. *P* ≤ 0.05 was considered statistically significant.

## Results

### Establishment of PDGAX Mouse Models

**Fig. 1:**
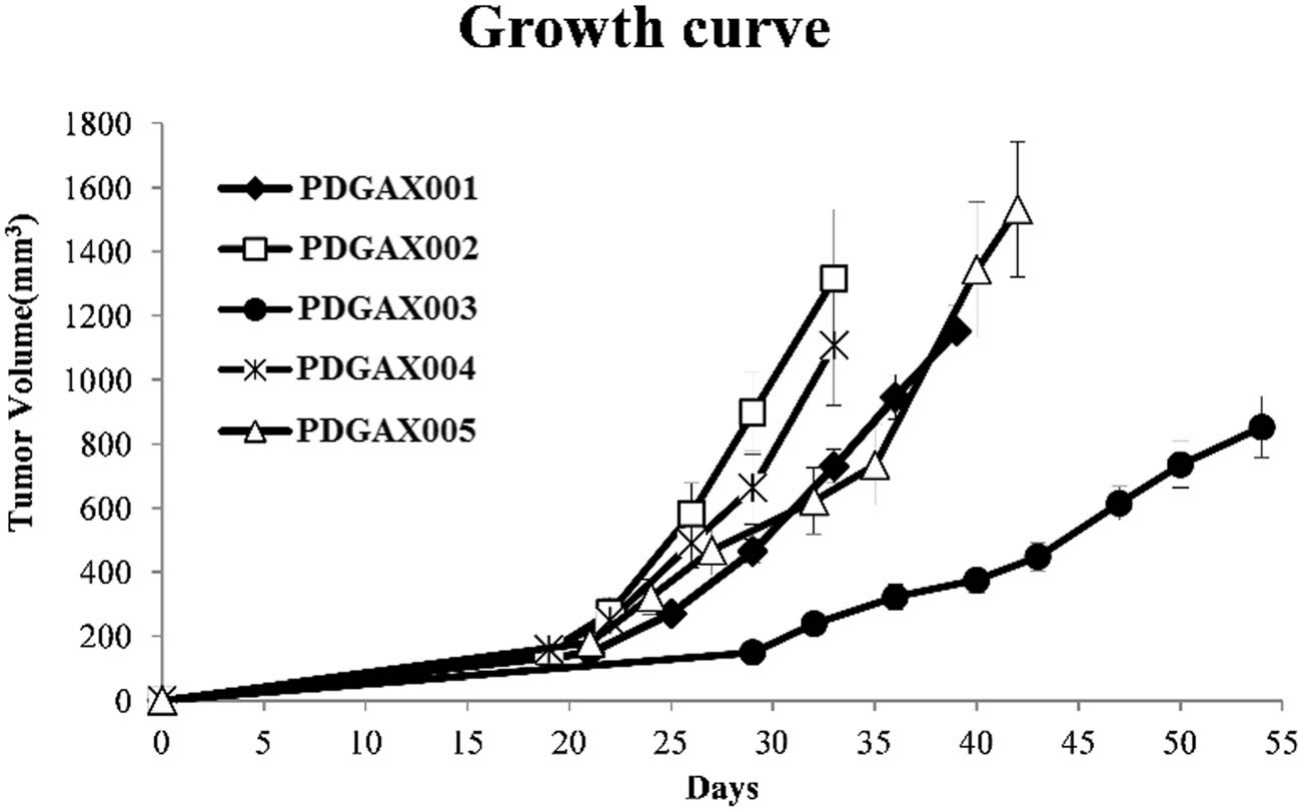
PDGAX model tumor growth curves. All five patient-derived gastric adenocarcinoma samples showed growth after subcutaneous implantation in SCID mice and subsequently in nude mice

Age, gender and tumor characteristics from five GA patients were collated (Table 1). All five patients’ GA samples were viable and led to growth in SCID mice after subcutaneous implantation, and continued to grow in nude mice after the second generation. The growth curves of the five established PDGAX mouse models are shown in Fig. 1. Autopsy examination of all PDGAX tumor-bearing mice at 2–3 months post-implantation revealed no evidence of metastases in the stomach, brain, lung, liver or kidney.

**Table 1:** Patient and PDGAX mouse model information

Patient information				Characterization & antitumor efficacy in the patient-derived GA xenograft models										
					HER-2		Mutation						Anti-tumor	
													Efficacy (TGI%)	
Gender/age	Pathology	TNM stage	Grade	Model ID	FISH	IHC	AKT1	FGFR4	PI3KCA	PTCH	PTEN	TP53	Trastuzumab	P value
M/57	AC	III	III	PDGAX001	AMP	+++	WT	WT	WT	WT	WT	Mutated	19	0.0904
M/54	AC	IV	III	PDGAX002	Non AMP	++	WT	WT	WT	WT	WT	WT	0	0.3318
M/51	AC	II	II	PDGAX003	Non AMP	+++	WT	WT	WT	WT	WT	WT	26	0.0626
M/58	AC	IV	III	PDGAX004	Non AMP	–	WT	WT	WT	WT	WT	WT	9	0.3309
F/62	AC	III	III	PDGAX005	AMP	+++	WT	WT	WT	WT	WT	WT	107	<0.0001

*M* male, *F* female, *AC* adenocarcinoma

### Histology and Tumor Grade

**Fig. 2:**
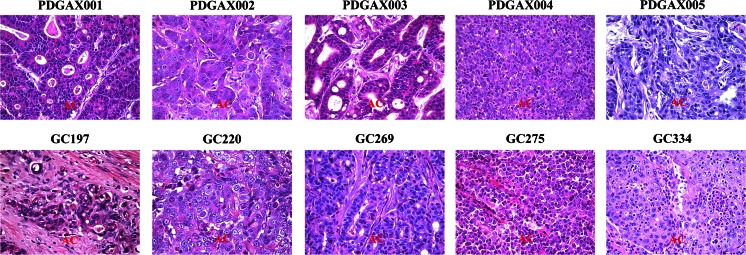
Histological evaluation of the PDGAX mouse models and matched human primary tumors. Representative images showing similar histological features (adenocarcinoma) between each PDGAX mouse model (*top row*) and the corresponding patient GA tissue (*bottom row*). AC: adenocarcinoma

All five PDGAX mouse models and their corresponding patient’s GA tissues were classified as gastric adenocarcinoma by pathologist histological evaluation. Similar histological features were observed between each xenograft model and its corresponding patient GA tissue (Fig. 2).

### Characterization of HER-2 Protein and Gene Expression in PDGAX Models

**Fig. 3:**
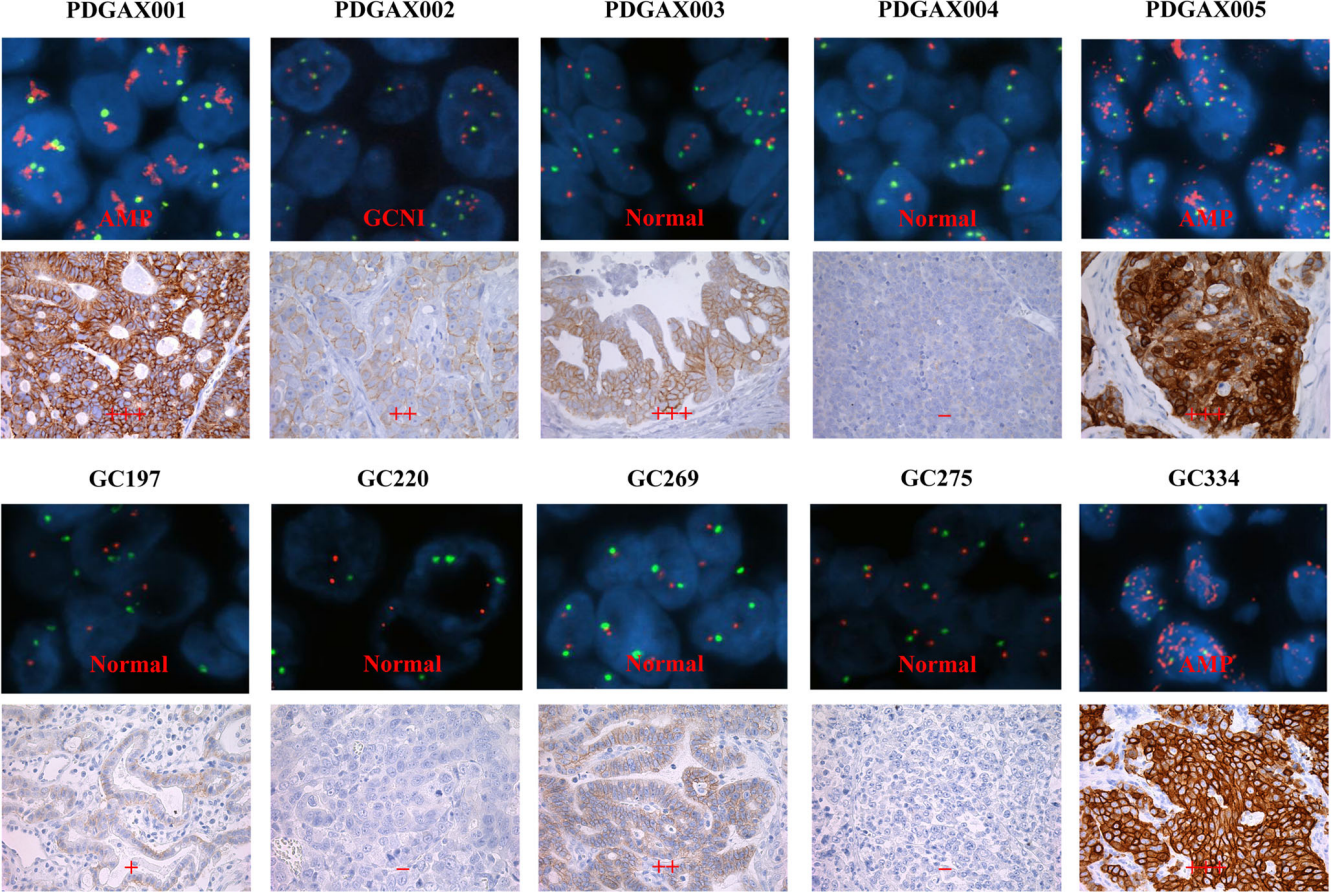
HER-2 IHC and FISH analysis. Representative images showing HER-2 IHC and FISH results on PDGAX mouse models (*top row*) and matched human primary tumor tissues (*bottom row*). HER-2 strong staining (3+) and FISH amplification (AMP) were detected in models PDGAX005 and PDGAX001 and matched primary tumor tissues. PDGAX001, PDGAX002, PDGAX003, and PDGAX004 were shown to be HER-2 IHC (0 ~ 3+) and FISH non-AMP. For FISH data, red signals represent HER2 and green signals represent CEP17

Following Hoffman’s HER-2 scoring criteria [16], three PDGAX mouse models (PDGAX001, PDGAX003 and PDGAX005) were scored as strongly positive for HER-2 membrane staining (3+), one model (PDGAX002) was scored as moderately positive (2+), and one model (PDGAX004) was scored as HER-2 negative (Fig. 3). According to Hoffman’s criteria, the scoring of *HER-2* gene copy number determined by FISH assay was ‘score 6’ (gene amplification) for models PDGAX001 and PDGAX005, ‘score 5’ for model PDGAX002, and ‘score 2’ for models PDGAX003 and PDGAX004. However, only model PDGAX005 showed a gene copy number increase with an average copy number of more than 20, whereas the average gene copy numbers of the other models were less than 6.0. In addition, all five PDGAX mouse models and their corresponding patient GA tissues showed a highly similar pattern of HER-2 protein expression and gene copy number (Fig. 3).

### Antitumor Efficacy of Trastuzumab in PDGAX Mouse Models

**Fig. 4:**
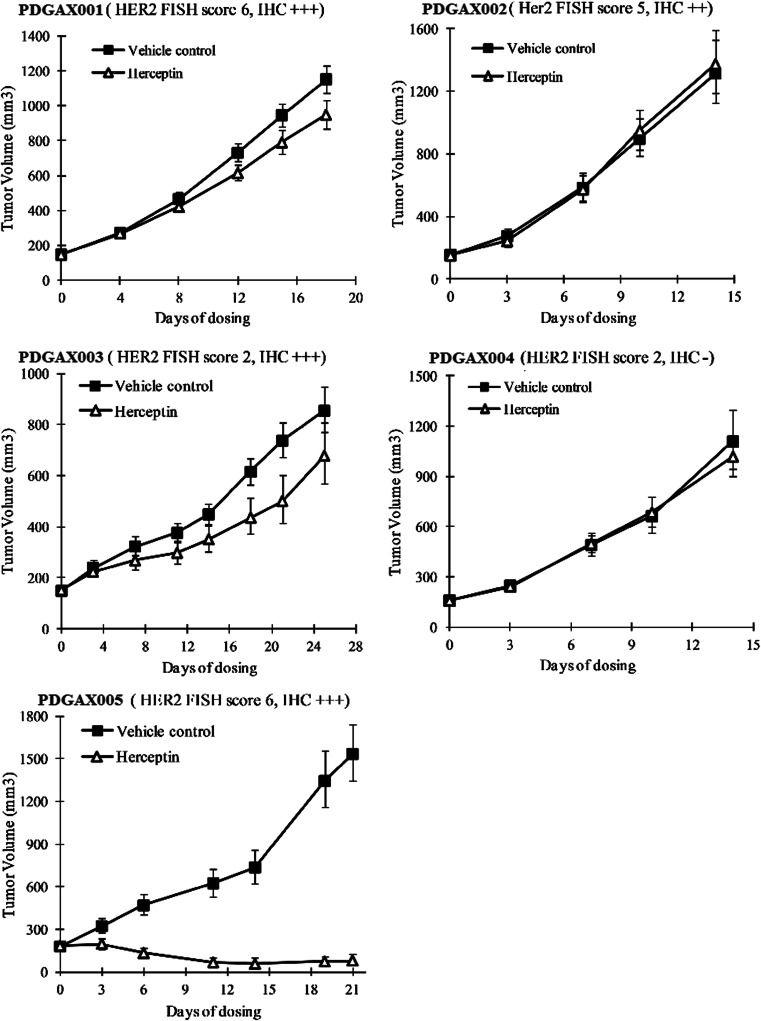
Trastuzumab anti-tumor efficacy in PDGAX models. Xenograft tumors from stable passages of the PDGAX models were implanted subcutaneously into nude mice and treated with vehicle control or trastuzumab 15 mg/kg twice a week for two or 4 weeks, based on tumor growth rates in the different models when tumors reached 150 ~ 250 mm^3^ post implantation. Significant tumor regression was only observed in model PDGAX005 (TGI 105 %), while moderate or no responses (0 ~ 26 % TGI) were observed in PDGAX001, PDGAX002, PDGAX003 and PDGAX004 models. Tumor volumes were measured twice a week. Tumor growth inhibition (%TGI) was calculated based on tumor volumes of the control and treatment groups

Trastuzumab anti-tumor efficacy studies were performed using a twice per week dosing schedule (15 mg/kg, i.p.) in all five PDGAX mouse models (Fig. 4 and Table 1). Three PDGAX models (PDGAX002, 003, and 004), which did not fit the current eligibility criteria for trastuzumab, showed either no or partial responses to trastuzumab treatment (PDGAX002, 0 % TGI, *P* = 0.3321; PDGAX003, 26 % TGI, *P* = 0.0627; PDGAX004, 9 % TGI, *P* = 0.3303). As expected, in model PDGAX005 (HER2 FISH AMP, IHC 3+, average gene copy number > 20), treatment with trastuzumab induced significant tumor regression (105 % TGI, *P* < 0.0001). Surprisingly, however, model PDGAX001 (HER2 FISH AMP, IHC 3+) failed to respond to trastuzumab (19 % TGI, *P =* 0.0904), despite evidence of strong HER2 protein expression and fulfillment of the clinical trastuzumab eligibility criteria. To further explore this finding, we sought to detect gene mutations that may confer resistance to trastuzumab.

### Pharmacodynamic Modulation of pHER2 by Trastuzumab

**Fig. 5:**
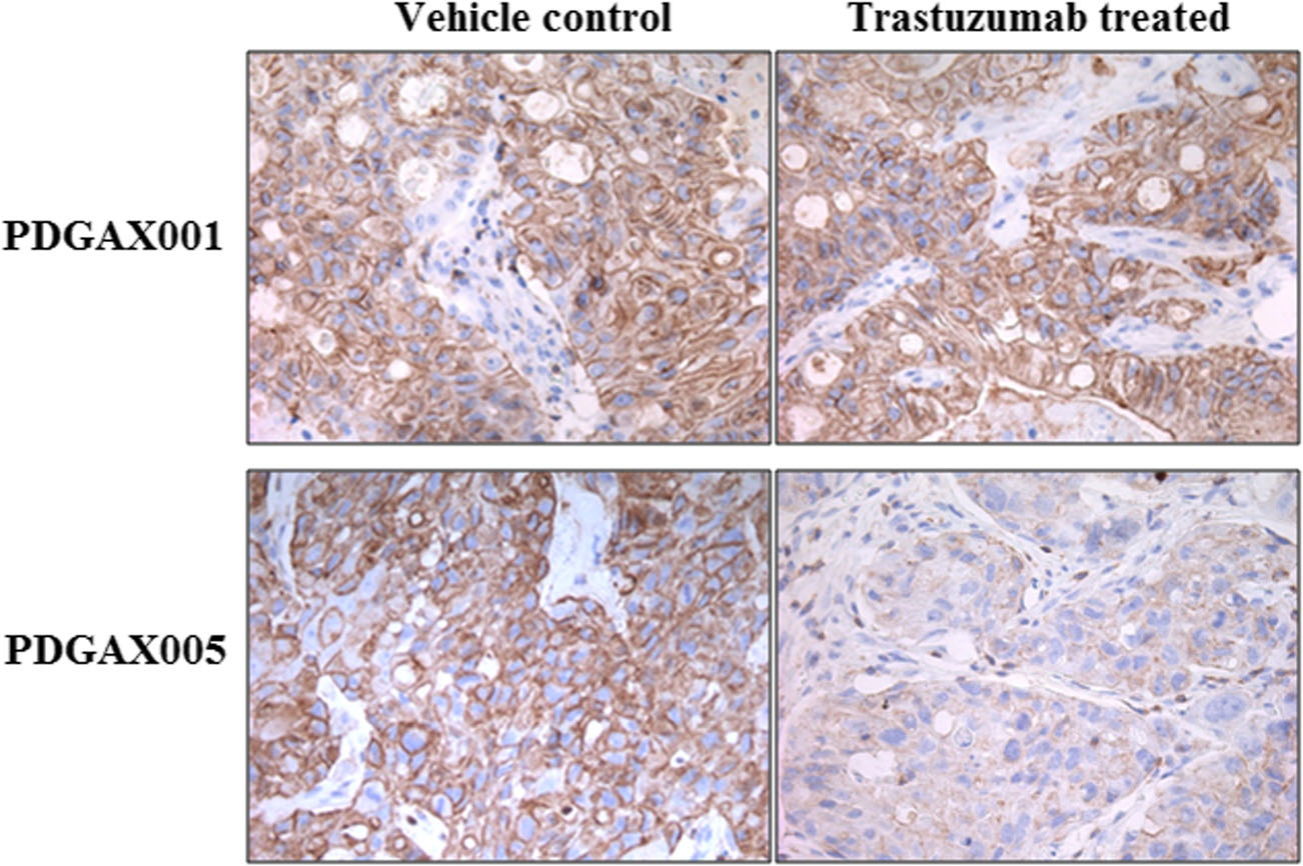
Detection of pHER2 IHC modulation in models PDGAX001 and PDGAX005. Representative images showing pHER2 modulation in trastuzumab-treated tissues from model PDGAX005 compared with vehicle control group. No modulation observed in model PDGAX001

pHER2 IHC was performed on vehicle control and trastuzumab treatment groups from PDGAX001 and PDGAX005 efficacy studies, both of which were HER2 IHC 3+ and HER2 FISH AMP, but displayed very different responses to trastuzumab. As expected, clear pHER2 modulation was observed in trastuzumab-treated tissues from model PDGAX005, but not in model PDGAX001 (Fig. 5), indicating that pharmacodynamic modulation of pHER2 correlates to trastuzumab response in model PDGAX005.

### Gene Mutation Screening

**Fig. 6:**
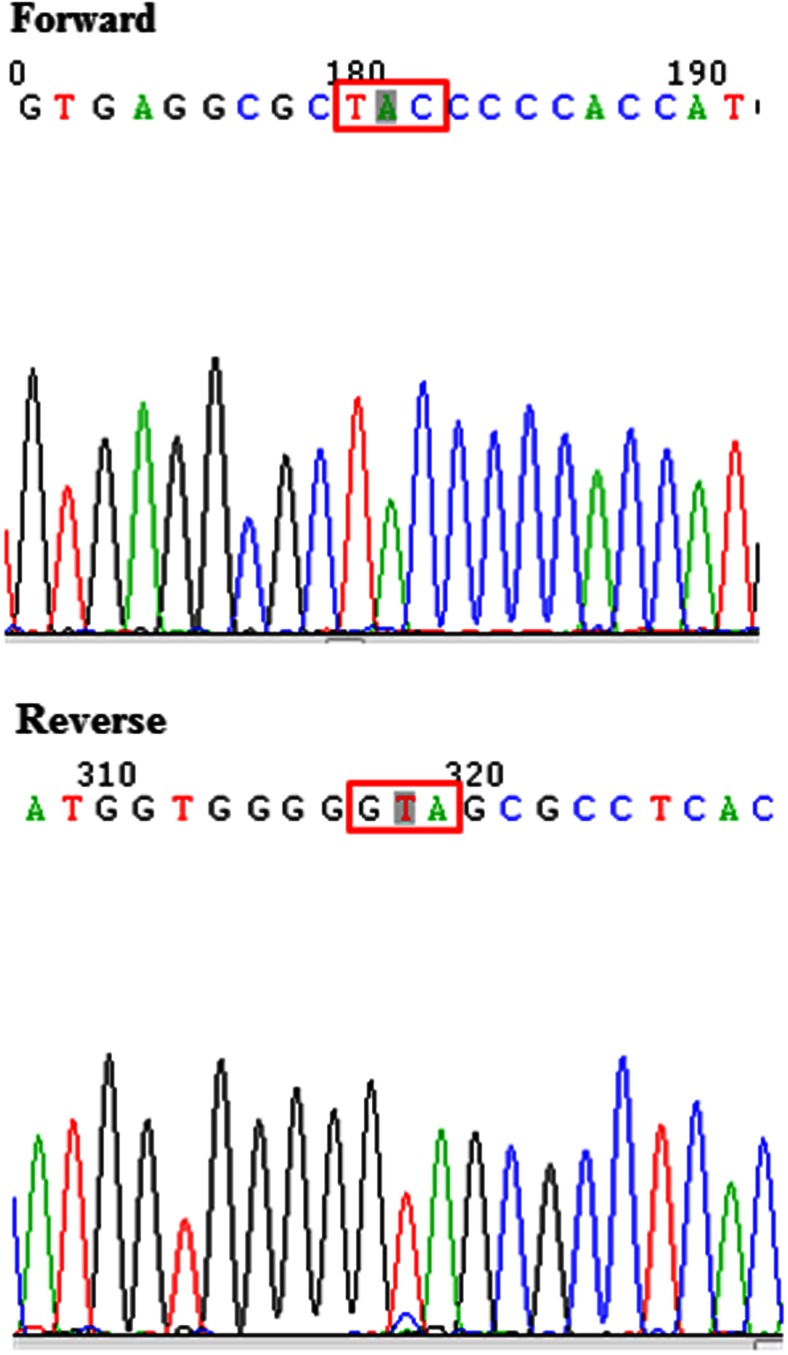
TP53 gene mutation detected by PCR-direct sequencing. An illustration of the mutation (G - > A) identified in TP53 gene (both on forward and reverse sequences). This mutation led to an amino acid change from cysteine to tyrosine at the 176th peptide of TP53 protein

A panel of genes including; *AKT1, FGFR4, PIK3CA, PTCH, PTEN, TP53* were screened in all of the PDGAX mouse models and corresponding patient GA tissues. Interestingly, the *TP53* mutation was identified in exon 5 from both the PDGAX001 model and the corresponding patient GC tissue. This was subsequently confirmed by direct sequencing (Fig. 6). A mutation (G - > A) was identified which causes an amino acid change from cysteine to tyrosine at the 176th peptide of TP53 protein. The functional meaning of this mutation is unclear. No other mutations were detected in the PDGAX models or their corresponding patient GA tissue.

## Discussion

Considering the current economic and ethical issues within modern drug discovery, relevant and predictive animal models of human cancer are immensely important in the search for new therapeutics. Subcutaneous tumor implantation has been the standard methodology in establishing animal models for human cancer research [20, 21]. However, standard xenografts make use of permanent tumor cell lines instead of primary tumors, and often have poor predictive value in determining human drug efficacy [22]. PDGAX models more closely resemble primary human tumors in terms of tissue architecture and molecular heterogeneity and thus offer the potential to better predict drug efficacy in humans [22]. To date, few PDGAX mouse models have been successfully established from GA patient tissues. Herein, we have successfully created five PDGAX mouse models by directly implanting fresh GA patient tissues into immune deficient mice. All five PDGAX mouse models and their corresponding patients’ gastric cancer tissues displayed a highly similar pattern in HER-2 protein expression and gene copy number. This finding gives further support to the hypothesis that PDGAX mouse models can more accurately represent clinical disease. Furthermore, we have used these PDGAX mouse models to successfully evaluate the efficacy of trastuzumab in gastric adenocarcinoma.

An excellent correlation between trastuzumab efficacy and HER-2 expression/ amplification has been shown in advanced gastric and gastroesophageal junction (GEJ) adenocarcinoma patients by the ToGA trials [1] and other previous studies [23]. According to the latest clinical practice guidelines [1, 14, 24], trastuzumab, in combination with cisplatin and a fluoropyrimidine, is recommended for advanced HER-2 positive GC. For clinicians, accurate and reliable identification of patients eligible for HER-2 targeted therapy is of crucial importance. The algorithm proposed by Hoffmann [16] incorporates a combination of IHC and FISH analyses and is the most appropriate HER-2 scoring system currently in use for the identification of trastuzumab-eligible gastric cancer patients. HER-2 positivity is defined as either IHC positive (3+) or FISH positive (HER-2/CEN-17 ≥ 2.2 or HER-2 gene copy number ≥ 6). However, according to the results of the ToGA trial, there is little difference in the proposed algorithm for HER-2 testing in gastric cancer between the FDA in the US and the European Medicines Agency [14]. Furthermore, there is no apparent benefit in patients with IHC 0 to 1+/FISH positive disease, suggesting that an IHC assay may be better suited to the selection of patients for treatment with trastuzumab [14]. In our study, two models (PDGAX001 and PDGAX005) were identified as HER-2 positive by IHC3+ and FISH AMP. Our data demonstrated significant tumor regression after treatment with trastuzumab in model PDGAX005 model (with a higher average gene copy number), but not in PDGAX001 model (with a lower average gene copy number).

To explore the mechanism underlying the difference in trastuzumab anti-tumor efficacy between the two HER-2 IHC 3+ and FISH AMP PDGAX models (PDGAX005 and PDGAX001), molecular genetic studies were performed. After screening gene mutations in *AKT1, FGFR4, PIK3CA, PTCH, PTEN* and *TP53* in the 5 PDGAX mouse models and their corresponding primary tumors, mutation of *TP53* was identified in model PDGAX001 and the corresponding patient tissue (Fig. 4 and Table 1). This result suggested that *TP53* mutation might represent a mechanism through which trastuzumab resistance may be conferred. Further work is ongoing to explore the mechanism underneath.

In summary, we have established novel xenograft models derived from patient gastric adenocarcinoma tissues and used these clinically relevant animal models to investigate the correlation of trastuzumab anti-tumor efficacy with HER-2 expression. Our results showed that PDGAX models could accurately recapitulate trastuzumab clinical data in the preclinical setting, underscoring their predictive power for future drug discovery.
